# Effect of Zhizhu Kuanzhong capsule on functional dyspepsia: Protocol for a systematic review and meta-analysis of randomized controlled trials

**DOI:** 10.1097/MD.0000000000009731

**Published:** 2018-02-09

**Authors:** Haixiong Lin, Xiaotong Wang, Xiuting Du, Junyue Wang, Yusi Li, Ren Zhang

**Affiliations:** a The First School of Clinical Medicine, Guangzhou University of Chinese Medicine, Guangzhou; b Shenzhen Bao’an Traditional Chinese Medicine Hospital Group, Guangzhou University of Chinese Medicine, Shenzhen; c The Second School of Clinical Medicine; d The College of Fundamental Medical Science, Guangzhou University of Chinese Medicine, Guangzhou, People's Republic of China.

**Keywords:** functional dyspepsia, protocol, systematic review, Zhizhu Kuanzhong capsule

## Abstract

**Background:**

Functional dyspepsia (FD) is a gastrointestinal disorder affecting people in the globe. Spleen-deficiency syndrome is one of its basic syndromes. Zhizhu Kuanzhong capsule was the most frequent Chinese herbal formula used in the spleen-deficiency researches. We aim to assess the effectiveness and safety of Zhizhu Kuanzhong capsule on FD.

**Methods:**

A systematic literature search for randomized controlled trials (RCTs) from their inception until December 31, 2017 will be conducted using 7 databases: PubMed, Cochrane Library, Embase, VIP Database, Chinese National Knowledge Infrastructure, Wanfang Data, and Chinese BioMedical Database. Inclusion the trials of Zhizhu Kuanzhong capsule which evaluate motilin level, clinical syndrome, and side effect in people with FD. The primary outcome measures will be motilin level, clinical syndrome integral, and clinical total effective rate. Data extraction and risk of bias assessments will be performed by 2 reviewers independently. Methodological and reporting quality of included studies will be assessed by the consolidated standards of reporting trials for Chinese herbal medicine formulas. All statistical analyses will be conducted using RevMan V.5.3 software. Funnel plots, Begg's test, and Egger's test will be developed to evaluate reporting bias.

**Results:**

This review will assess the effect of Zhizhu Kuanzhong capsule on clinical total effective rate, symptom remission rate, plasma motilin level, and safety of FD in patients.

**Conclusion:**

The conclusion of our study will provide updated evidence to judge whether Zhizhu Kuanzhong capsule is an effective intervention for patients with FD.

## Abstract 2

Supplemental Digital Content is available in the text

## 1 Introduction

FD is a common condition in clinical practice affecting people in both eastern and western parts of the globe, especially in Asia, Europe and North America. According to the Roman III standard, the prevalence has been declared to range from 5.3% to 20.2%.^[1]^ The symptoms of FD are mainly manifested in epigastric pain or burning, early satiation, and postprandial epigastric fullness, which persisting for at least 3 months.^[2]^ However, routine diagnostic tests, including endoscopy, could not find any pathology to explain these symptoms.^[2]^ People with FD have a poorer mood, poorer sleep quality, and reported a mean of 1.4 hours absence from work within a week.^[3,4]^ FD not only leads to an impact on health related quality of life, but also leads to a significant economic burden on the healthcare system.^[4]^ A study reported that the costs of FD were conservatively estimated to be $18.4 billion in 2009 in the US population.^[4]^ Prokinetic agents, as primary pharmacological treatment for FD, could promote gastric emptying, alleviate the symptoms of patients with FD.^[5]^ A review reported that the relative risk of prokinetics was significantly reduced compared with placebo.^[6]^ However, in spite of some beneficial effects, prokinetics have adverse effects on central nervous and heart.^[7]^ Therefore, many patients also seek alternative therapies to manage their gastrointestinal discomfort, including acupuncture and Chinese herbal medicine (CHM).^[8,9]^

According to the theory of Chinese medicine, FD is divided into different syndromes base on various clinical symptoms and signs.^[10]^ Spleen-deficiency syndrome is one of its basic syndromes.^[10]^ Zhizhu Kuanzhong capsule was the most frequent Chinese herbal formula used in the spleen-deficiency researches.^[11]^ One systematic review suggested that CHM, such as liu jun zi tang, or xiang sha liu jun zi tang, was promising for managing FD.^[8]^ However, no systematic review regarding Zhizhu Kuanzhong capsule in FD has been planned or published yet. In addition, there is insufficient evidence to support the widespread use of Zhizhu Kuanzhong capsule. Therefore, the purpose of our study is to investigate current evidence related to the effectiveness and safety of Zhizhu Kuanzhong capsule as a treatment for FD.

## 2 Methods

### 2.1 Study type

We will include the RCTs that evaluated the clinical symptom integral, clinical total effective rate, motilin level, electrogastrogram, or side effect of Zhizhu Kuanzhong capsule in people with FD. No restrictions on race, region, sex, age, severity, or duration of patients with FD. However, some studies use the word randomization instead of describing the randomization method in detail in China. We will include such trials and assess the risk of bias as high unless detailed randomization processes are described. In addition, we will exclude some trials that used inappropriate randomization processes, such as the order of hospitalization, tossing of a coin. Studies of reviews, cross-sectional, cohort, animal experiments, and comments will not be included.

### 2.2 Participants

Patients with FD will be considered in the systematic review. The diagnostic criteria for FD in the trials is in accordance with the Rome III standards or Rome IV standards, which including the symptoms of postprandial fullness, early satiation, epigastric pain or burning, etc.^[12]^ Patients with other types of diseases, such as severe cardiac dysfunction, severe hepatic dysfunction, severe kidney dysfunction, endocrine disease, cholecystitis, pancreatitis, and peptic ulcer, will not be included.

### 2.3 Interventions

Intervention group will receive Zhizhu Kuanzhong capsule, either as the sole treatment or as an adjunct to other treatment which were applied in both groups. Control group intervention could be conventional medication, no treatment, placebo, or prokinetics, such as domperidone, mosapride, cisapride, etc.

### 2.4 Outcome measures

The primary outcome measures of motilin level, clinical syndrome integral, and clinical total effective rate will be measured. The criteria of clinical syndrome integral according to the severity of clinical symptoms and signs, such as nausea and vomiting, loss of appetite, epigastric discomfort, belch, heartburn, and acid reflux. The clinical syndrome scores are divided into 4 levels^[13]^: free, 0 points, no obvious clinical symptoms or signs. Light level: 1 point, relatively obvious clinical symptoms. Medium level: 2 points, relatively obvious clinical symptoms, and abdominal circumference increased, but had no obvious effect on the work. Severe level: 3 points, clinical symptoms and signs are very significant, and have a certain effect on the patient's normal work. At last, summarize the symptom integrals together. The criteria of clinical total effective rate will be calculated based on the following criterion^[13]^: the main symptoms and signs were significantly reduced, and the clinical syndrome integral was reduced by more than 60% after treatment.

The secondary outcome measures will be electrogastrogram and adverse events.

### 2.5 Data sources

The following databases will be searched from inception to December 31, 2017: PubMed, Cochrane Library, Embase, VIP Database, Chinese National Knowledge Infrastructure, Wanfang Data, and Chinese BioMedical Database. The search term will be composed of the intervention term part, disease term part, and study type term part: (“Zhizhu Kuanzhong capsule” or “Zhizhu Kuanzhong” or “Zhizhu Kuanzhong granule” or “Zhizhu Kuanzhong decoction” or “Zhizhu Kuanzhong formula” or “Zhizhu Kuanzhong tang” or “Zhizhu Kuanzhong pill” or “Zhizhu Kuanzhong tablet”) and (“functional dyspepsia” or “dyspepsia” or “indigestion” or “FD” or “gastrointestinal discomfort” or “gastrointestinal dysfunction” or “epigastric pain” or “epigastric burning” or “epigastric satiation” or “postprandial epigastric fullness”) and (“randomized controlled trial” or “randomized”) and (“blind”). The search strategies that will be applied to the PubMed and CNKI are presented in the Supplementary File 1. Similar search strategies will be conducted to the other databases. Publication languages will be limited to Chinese and English. Clinical trials published in abstract form will be selected if sufficient data could be retrieved from the abstract or authors. Reference lists of the potentially eligible studies will be reviewed to discover additional clinical trials. Treatment strategies that are not repeated will be deleted.

### 2.6 Study selection and data extraction

**Figure 1:**
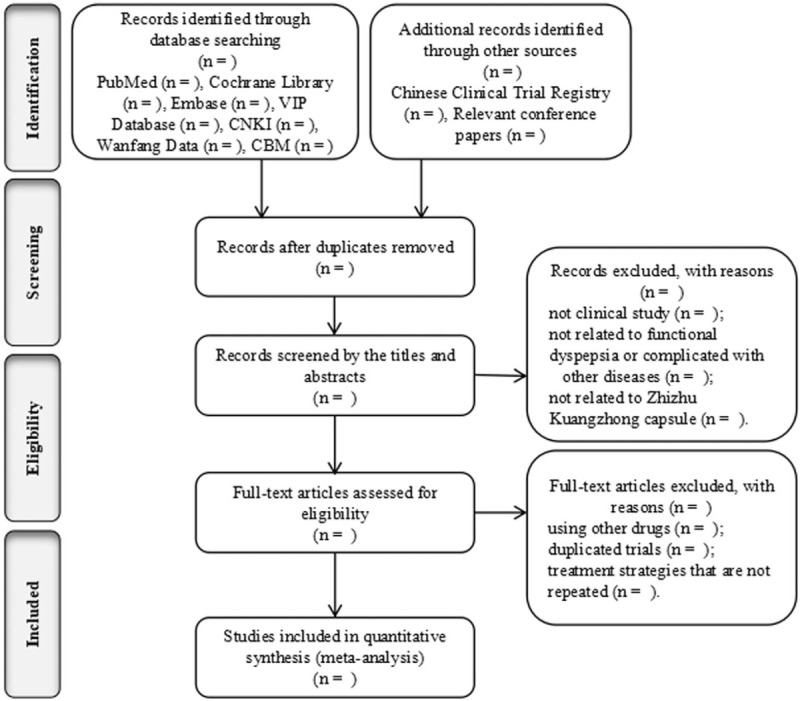
Flow chart of the search process.

We will import all retrieved results into NoteExpress 3.2.0. Duplicate data from different databases will be identified first. Two reviewers (HX Lin and XT Wang) independently will screen the remaining titles and abstracts to select potential trials, and then review full texts for eligible trials according to the criteria described above. The selection process will be showed in a PRISMA flow chart (http://www.prisma-statement.org/) (Fig. 1). Two reviewers (HX Lin and XT Wang) will extract general information (first author names, publication year), study patients (age of participants, gender, sample sizes), therapeutic strategy (intervention methods and treatment duration), dropout number, outcome, and follow-up periods. Disagreements will be resolved through discussed or consultation with a third author (R Zhang).

### 2.7 Addressing missing data or unclear measurement scales

Additional information or missing data will be acquired by contacting the original authors via telephone or email. If impossible, we will analyze the available data from the study and take into account the potential impact of insufficient information on the review results in the Discussion Section.

### 2.8 Risk of bias in included studies

Risk assessment and quality evaluation, as developed by the Cochrane, will be used to assess each eligible study. This risk assessment including random sequence generation, allocation concealment, blinding of participants and personnels, blinding of outcome assessments, incomplete outcome data, selective reporting and other source of bias. Finally, the risk will be classified into three categories: high, unclear, and low.

### 2.9 Data synthesis and analysis

We will analyze the included data using RevMan V.5.3 software. Data will be pooled and expressed as mean difference or standardized mean difference for continuous outcomes and risk ratio for dichotomous outcomes with 95% confidence interval (CI) using random or fixed effects models. Statistical heterogeneity will be evaluated using the Chi-square test and *I*^2^ values.^[14]^*I*^2^ > 75% indicate significant heterogeneity, 50% < *I*^2^ ≤ 75% is regarded as mild heterogeneity, and 0% ≤ *I*^2^ ≤ 50% is considered no heterogeneity.^[15]^ A random-effect model will be performed when mild or significant heterogeneity among the studies (*P* < .05, *I*^2^ > 50%) occurred among the studies. Methodological and reporting quality of included studies will be assessed by the consolidated standards of reporting trials for Chinese herbal medicine formulas (CONSORT-CHM Formulas).^[16]^

### 2.10 Additional analyses

Meta regression and subgroup analysis will be performed to explore source of heterogeneity. Sensitivity analysis will be conducted to identify the robustness of the result by excluding low-quality trials. subgroup analysis will be performed based on various study characteristics, such as study quality, study location, intervention methods, treatment duration. Qualitative synthesis will be performed if the data extraction is insufficient.

### 2.11 Assessment of reporting biases

Funnel plots will be performed to evaluate the potential reporting bias. If asymmetry is showed by a visual inspection, we will perform Begg's test and Egger's test. Interpret values of *P* > .05 in Begg's test or Egger's test as showing no significant publication bias.

### 2.12 Quality of evidence

The quality of evidence of the included studies will be evaluated for relevant outcomes using the approach of the Grading of Recommendations Assessment, Development and Evaluation (GRADE).^[17]^ The limitations of the clinical study, inconsistencies, indirect evidence, inaccuracies, and publication bias will be considered. The quality of the evidence will be divided into 4 levels: very low, low, moderate, or high.

### 2.13 Ethics and dissemination

We aim to explore the clinical effectiveness and safety of Zhizhu Kuanzhong capsule on patients with FD, especially motilin level, clinical syndrome integral, clinical total effective rate. At last, this study will be published in a peer-reviewed journal. This study does not require ethical approval because only published data will be included. The result will be submitted to a peer-reviewed journal.

## 3 Discussion

FD is a gastrointestinal disorder characterized by clinical symptoms thought to originate in the upper abdomen without an identifiable organic cause, or metabolic abnormalities that readily explains them.^[18]^ Numerous factors contribute to the symptomatology of FD, such as disturbed gastric motility, gastric sensation, or gastric and duodenal inflammation.^[19]^ According to the current guidelines and expert consensus recommendation, prokinetics is one of the routine treatments for FD.^[20]^ However, the efficacy of prokinetics is still not satisfactory, and with potential side effects in extrapyramidal reactions, cardiac arrhythmic.^[7]^

A survey reported that the treatment of functional gastrointestinal disorders was based on symptoms relieved by drugs.^[21]^ Chinese medicine is widely applicable in the treatment of FD by utilizing a typical symptoms-based approach.^[22]^ Several systematic reviews have already been published suggested that CHM was more effective than gastrointestinal prokinetic drug for patients with FD.^[23,24]^ To our knowledge, even though Zhizhu Kuanzhong capsule is frequently used in FD,^[9]^ no previous relevant systematic review on the effects of Zhizhu Kuanzhong capsule on FD has been planned or published yet. Zhizhu Kuanzhong capsule is composed of baizhu (Atractylodes macrocephala Koidz.), zhishi (Citrus aurantium L.), chaihu (Bupleurum chinense), shanzha (Crataegus pinnatifida).^[25]^ Previous studies have indicated that Zhizhu Kuanzhong capsule could fortify the spleen and soothe the liver, which used in the spleen-deficiency.^[11]^ However, The effectiveness and safety of Zhizhu Kuanzhong capsule in the treatment of FD has not been fully elucidated. The purpose of this review is to assess the effect of Zhizhu Kuanzhong capsule on clinical symptom integral, clinical total effective rate, motilin level, electrogastrogram, and safety of FD in patients. In particular, we will identify specific treatment strategies that are used in FD according to the theory of Chinese medicine. In order to ensure the accuracy and reliability of the results. Intervention methods that are not repeated will be eliminated. We are intended to use sufficient evidence to ensure adequate power for this meta-analysis. Herein, this systematic review will be the first to evaluate the clinical efficacy and safety of Zhizhu Kuanzhong capsule in patients with FD, and may benefit practitioners in the fields of traditional and conventional medicine.

## 4 Author contributions

Conceived the idea for this study and drafted the protocol; developed the search strategies, conducted data collection, and analyzed independently: HX Lin and XT Wang.

Revised the manuscript: R Zhang.

Performed risk of bias assessment: XT Du, JY Wang, and YS Li.

Supervise the project: R Zhang.

Approved the final manuscript: All authors.

## Acknowledgements

The authors thank Dr YaLi Huang and Ren Zhang from Guangzhou University of Chinese Medicine for their valuable suggestions on the manuscript. In addition, HaiXiong Lin thanks the inimitable care and support of XiaoTong Wang over the years. I love you. Will you spend the rest of your life with me?

## Supplementary Material

Supplemental Digital Content
