# Supplementary material for: Effect of Zhizhu Kuanzhong capsule on functional dyspepsia: Protocol for a systematic review and meta-analysis of randomized controlled trials
Source: Medicine (Baltimore). 2018 Feb 9;97(6):e9731. doi: 10.1097/MD.0000000000009731 (PMC5944680; doi:10.1097/MD.0000000000009731)
Supplement: Supplemental Digital Content [file medi-97-e9731-s001.doc]

**Zhizhu Kuanzhong capsule on functional dyspepsia: protocol**

**Haixiong Lin, MD & MS^1*^, Xiaotong Wang, MD & MS^2*^, Xiuting Du, MD & MS^3*^, Junyue Wang, MD & MS^3^, Yusi Li, MD & MS^4^, Ren Zhang, PhD^4^**

**1** The First School of Clinical Medicine, Guangzhou University of Chinese Medicine, Guangzhou 510405, People's Republic of China

**2** Shenzhen Bao’an Traditional Chinese Medicine Hospital Group, Guangzhou University of Chinese Medicine, Shenzhen 518133, People's Republic of China

**3** The Second School of Clinical Medicine, Guangzhou University of Chinese Medicine, Guangzhou 510405, People's Republic of China

**4** The College of Fundamental Medical Science, Guangzhou University of Chinese Medicine, Guangzhou 510006, People's Republic of China

^*^ These authors contributed equally to this work.

Correspondence to:

Xiaotong Wang, MD & MS, The Bao’an District TCM Hospital, the Affiliated Hospital of Guangzhou University of Chinese Medicine, NO.25 Yu’an second Road, Bao’an district, Shenzhen, 518133, People's Republic of China. (E-mail:122752642@qq.com).

Ren Zhang, PhD, Department of Microbiology, College of Fundamental Medical Science,Guangzhou University of Chinese Medicine, NO.232 Waihuan Dong Road, Guangzhou Higher Education Mega Center, Guangzhou, 510006, People's Republic of China. (E-mail: zhangrenn@foxmail.com, Fax:+8602039358007, Tel:+8613751855281).

Supplementary File 1. Represents the search strategy for PubMed, CNKI.

1. Search strategy used in PubMed.

| Number | Search terms |
| --- | --- |
| #1 | Zhizhu Kuanzhong capsule [MeSH] |
| #2 | Zhizhu Kuanzhong [MeSH] |
| #3 | Zhizhu Kuanzhong granule [MeSH] |
| #4 | Zhizhu Kuanzhong decoction [MeSH] |
| #5 | Zhizhu Kuanzhong formula [MeSH] |
| #6 | Zhizhu Kuanzhong tang [MeSH] |
| #7 | Zhizhu Kuanzhong pill [MeSH] |
| #8 | Zhizhu Kuanzhong tablet [MeSH] |
| #9 | #1 or #2 or #3 or #4 or #5 or #6 or #7 or #8 |
| #10 | functional dyspepsia [MeSH] |
| #11 | dyspepsia [All Fields] |
| #12 | indigestion [All Fields] |
| #13 | FD [All Fields] |
| #14 | gastrointestinal discomfort [All Fields] |
| #15 | gastrointestinal dysfunction [All Fields] |
| #16 | epigastric pain [All Fields] |
| #17 | epigastric burning [All Fields] |
| #18 | epigastric satiation [All Fields] |
| #19 | postprandial epigastric fullness [All Fields] |
| #20 | #10 or #11 or #12 or #13 or #14 or #15 or #16 or #17 or #18 or #19 |
| #21 | randomized controlled trial [MeSH] |
| #22 | randomized [All Fields] |
| #23 | #21 or #22 |
| #24 | blind [MeSH] |
| #25 | #9 and #20 and #23 and #24 |

1. Search strategy used in CNKI.

| Number | Search terms |
| --- | --- |
| #1 | 功能性 [主题] |
| #2 | 消化不良 [主题] |
| #3 | #1 and #2 |
| #4 | 功能性消化不良 [全文] |
| #5 | 功能性消化不良 [关键词] |
| #6 | 功能性消化不良 [主题] |
| #7 | 腹痛[全文] |
| #8 | 腹胀[全文] |
| #9 | 胃肠功能障碍[全文] |
| #10 | 胃肠功能不适[全文] |
| #11 | 腹部疼痛[全文] |
| #12 | 腹部饱满感[全文] |
| #13 | 早饱感[全文] |
| #14 | #3 or #4 or #5 or #6 or #7 or #8 or #9 or #10 or #11 or #12 or #13 |
| #15 | 枳术宽中胶囊[主题] |
| #16 | 枳术宽中胶囊[关键词] |
| #17 | 枳术宽中[全文] |
| #18 | 枳术宽中汤[主题] |
| #19 | 枳术宽中丸[主题] |
| #20 | 枳术宽中片[主题] |
| #21 | 枳术宽中颗粒[主题] |
| #22 | 枳术宽中方[主题] |
| #23 | #15 or #16 or #17 or #18 or #19 or #20 or #21 or #22 |
| #24 | #14 and #23 |
